# A Safer Mosquito Treatment?: Minimizing Deltamethrin Risks to Children

**Published:** 2005-06

**Authors:** David J. Tenenbaum

Indoor spraying to control disease-carrying mosquitoes is the strategy of choice in Mexico’s effort to reduce malaria. When Mexico discontinued the use of DDT for this purpose in 2000, the pyrethrum-derived compound deltamethrin became the primary pesticide in the battle against mosquitoes. To find out how deltamethrin is distributed, absorbed, and excreted, and how it affects human DNA, researchers at the Universidad Autónoma in San Luis Potosí, Mexico, tracked 32 Mexican children aged 3–12 years before and after their homes were sprayed with deltamethrin **[*EHP*** **113:782–786]**. Their findings suggest that with appropriate precautions, health risks to children exposed to deltamethrin can be minimized.

Deltamethrin is recommended by the World Health Organization for application to walls and mosquito nets, and is also used for other in-home insect control, and for agriculture. But during *in vitro* tests, deltamethrin has caused chromosome damage, which can be a precursor to cancer. There have also been published reports of neurotoxicity in exposed humans.

The children in the current study lived in four villages in the state of San Luis Potosí. The researchers sampled the soil of the homes’ dirt floors and measured metabolites of deltamethrin in the children’s urine at several time points in the 180 days after spraying. The urine metabolites served as biomarkers for systemic deltamethrin uptake.

The researchers also took blood samples from 28 children before spraying and then 24 hours afterwards, and looked for chromosome breaks using the comet assay. In this assay, cells are broken apart to remove proteins, and the DNA is allowed to unwind. When the DNA undergoes gel electrophoresis (separation in an electric field), DNA fragments move away, and damage is measured by counting the fragments that have migrated.

Half of the deltamethrin degraded in indoor soil within 2.2 weeks. Indoor soil levels peaked above 2 parts per million 8 days after spraying, and declined to about 0.5 parts per million at 180 days. The highest urine metabolite concentrations appeared within 24 hours of spraying, and 91% of the metabolites were excreted within 3 days. Metabolite concentrations had returned to undetectable prespraying levels after 180 days. The results of the comet assay were statistically identical between prespraying and postspraying blood samples, indicating no DNA damage resulting from exposure.

Although peaks in urine biomarkers did not correlate with those of deltamethrin measured in the dirt floors—allowing the researchers to dismiss soil ingestion as the most important pathway of exposure—the study did confirm that children in treated houses had higher levels of deltamethrin metabolites than children in the general population, as measured in previous studies. Other studies have shown limited absorption of pyrethroids through the skin. The researchers therefore suggest that inhalation in the first hours or days after spraying is the most important pathway of deltamethrin exposure for children.

The researchers conclude that children may be protected by keeping them out of sprayed areas for one day, and then cleaning cooking surfaces and utensils before use. In addition, children should be monitored to minimize soil ingestion or contact with sprayed walls.

## Figures and Tables

**Figure:**
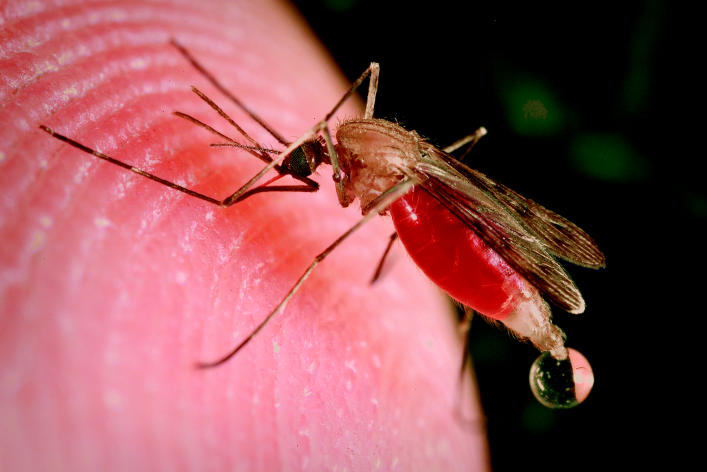
**Taking the health bite out of mosquito fighters.** Some simple precautions can protect children from the pesticide deltamethrin sprayed inside homes.

